# Human Rabies in China

**DOI:** 10.3201/eid1112.040775

**Published:** 2005-12

**Authors:** Yong-Zhen Zhang, Cheng-Long Xiong, Dong-Lou Xiao, Ren-Jie Jiang, Zhao-Xiao Wang, Ling-Zhu Zhang, Zhen F. Fu

**Affiliations:** * Chinese Center for Disease Control and Prevention, Beijing, China;; † Yancheng Municipal Center for Disease Control and Prevention, Yanchen, China;; ‡ Guizhou Center for Disease Control and Prevention, Guiyang, China;; § GuangxiCenter for Disease Control and Prevention, Nanning, China;; ¶ University of Georgia, Athens, Georgia, USA

**Keywords:** epidemiology, rabies, geographic distribution, letter

**To the Editor:** Rabies has occurred in China for >2,000 years and was first described in ≈56 BC (*1*). Since 1950, human rabies has been a class II notifiable disease in China, and the annual number and distribution of human rabies cases have been archived. We examined the archived data from 1950 to 2004 and analyzed epidemiologic characteristics.

During the 55-year period, 108,412 human rabies cases were recorded in China. The Figure shows the number of annual cases from 1950 to 2004; 3 major epidemics of human rabies in China are apparent. In the early 1950s, only a few cases occurred; the first peak occurred from 1956 to 1957 with ≈,000 cases each year. Then the number of cases declined during subsequent years and was relatively constant throughout the 1960s. By 1969, the number of cases increased again to ≈,000. This ascending phase continued throughout the 1970s and 1980s. The second epidemic peaked in the early 1980s. In 1981, 7,037 cases were recorded, the largest number of cases in a single year during the 55-year period. During the 1980s, 55,367 cases were reported (>5,000 cases annually), representing >50% of the 108,412 cases seen during the entire period. In the early 1990s, the number of human cases decreased dramatically from 3,520 in 1990 to 159 in 1996. However, this downward trend reversed its course in 1998, and annual cases have increased gradually since then. In 2004, a total of 2,651 cases were reported, an increase of >16 fold when compared with the numbers in 1996. This third rabies epidemic apparently has not yet peaked.

**Figure:**
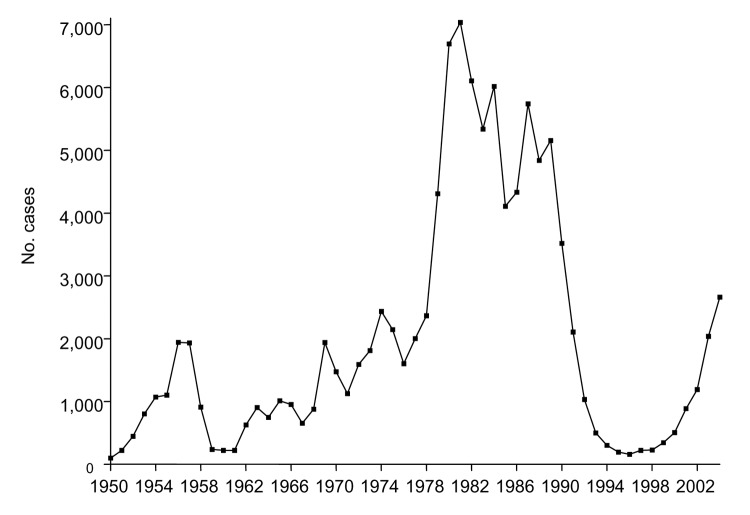
Annual rabies cases reported in China from 1950 to 2004.

The compiled data also showed substantially more rabies cases in the summer and autumn than in the spring and winter. Similar seasonality was reported in animals (*2*), indicating the pattern of transmission from animals to humans. Rabies patients range in age from infancy to >65 years of age. The ratio of male to female victims is 68 to 32. Although human rabies has been reported in almost all provinces, 15 provinces have had >1,000 cumulative cases each. These provinces are Hunan, Guangdong, Sichuan, Guangxi, Guizhou, Hubei, Jiangxi, Shandong, Henan, Anhui, Jiangsu, Hebei, Fujian, Yunnan, and Liaoning. These 15 provinces account for >93% of the total cases. Four provinces (Hunan, Guangdong, Sichuan, and Guangxi) have had >7,000 cumulative cases each.

Most of the human patients were infected with rabies by dog bites. The number of dogs has increased gradually in China since the late 1970s. Now ≈0% of households in Guangxi, Guizhou, and Jiangsu, and Hunan, where the most cases were recorded in recent years, have >1 dog (data not shown). However, the rate of dog vaccination remains ≈%.

The rabies epidemics in China since 1950 may be partially explained by dog population dynamics. The first major epidemic subsided at the end of the 1950s and the beginning of the 1960s, coinciding with pet reduction policy. The second major epidemic peaked in the late 1970s and early 1980s, when economic reforms were initiated in China and the dog population increased dramatically. Population immunity may also play a role in these cyclic epidemics. However, neither the dramatic decline of rabies cases in the early part of 1990s nor the initiation of the third epidemic around the turn of the millennium could be explained simply by dog population dynamics. Other factors may include untimely and inappropriate postexposure treatment (*3**,**4*) and the existence of healthy carrier dogs (*5**–**7*). Wounds of 118 of 178 patients were not treated; 60 of the patients washed the wounds with soap and water. A total of 129 (72%) patients did not receive vaccine. Of the remaining 49 (28%) patients, 35 received vaccination in a timely manner. Two of the 178 patients received antirabies serum intramuscularly. Among the 49 patients who received postexposure vaccination, 30 did not complete the immunization requirements. Healthy carriers have been detected, and rabies virus antigen was found in 25 (10%) of 248 brain specimens from healthy dogs collected from Guangxi, Guizhou, and Jiangsu (data not shown) in 1 study. Further investigation is needed to confirm these findings.

In summary, rabies remains a public health problem in China. Strategies to control and prevent human rabies include public education and awareness about rabies, pet vaccination programs, elimination of stray animals, and enhanced postexposure management. In addition, the large number of rabies cases should encourage rabies prophylaxis for foreign travelers before they visit China, particularly those who might travel to rural areas.
